# Proteomic analysis of serum proteins from HIV/AIDS patients with *Talaromyces marneffei* infection by TMT labeling-based quantitative proteomics

**DOI:** 10.1186/s12014-018-9219-8

**Published:** 2018-12-21

**Authors:** Yahong Chen, Aiqiong Huang, Wen Ao, Zhengwu Wang, Jinjin Yuan, Qing Song, Dahai Wei, Hanhui Ye

**Affiliations:** 1 Mengchao Hepatobiliary Hospital of Fujian Medical University, 312 Xihong Road, Fuzhou, 350025 Fujian Province People’s Republic of China; 2 Fuzhou Infectious Disease Hospital, Fujian Medical University, 312 Xihong Road, Fuzhou, 350025 Fujian Province People’s Republic of China; 3 The First Affiliated Hospital of Jiaxing University, 1882 Zhonghuan Road, Jiaxing, 314001 People’s Republic of China; 4 Department of Immunology and Microbiology, Shanghai Jiao Tong University School of Medicine, 280 South Chongqing Road, Shanghai, 200025 People’s Republic of China; 5 Shanxi Institute of Flexible Electronics, Northwestern Polytechnical University, 127 West Youyi Road, Xi’an, 710072 People’s Republic of China

**Keywords:** Quantitative proteomics, HIV/AIDS, *Talaromyces marneffei*, IL1RL1, THBS1

## Abstract

**Background:**

*Talaromyces marneffei* (TM) is an emerging pathogenic fungus that can cause a fatal systemic mycosis in patients infected with human immunodeficiency virus (HIV). Although global awareness regarding HIV/TM coinfection is increasing little is known about the mechanism that mediates the rapid progression to HIV/AIDS disease in coinfected individuals. The aim of this study was to analyze the serum proteome of HIV/TM coinfected patients and to identify the associated protein biomarkers for TM in patients with HIV/AIDS.

**Methods:**

We systematically used multiplexed isobaric tandem mass tag labeling combined with liquid chromatography mass spectrometry (LC–MS/MS) to screen for differentially expressed proteins in the serum samples from HIV/TM-coinfected patients.

**Results:**

Of a total data set that included 1099 identified proteins, approximately 86% of the identified proteins were quantified. Among them, 123 proteins were at least 1.5-fold up-or downregulated in the serum between HIV/TM-coinfected and HIV-mono-infected patients. Furthermore, our results indicate that two selected proteins (IL1RL1 and THBS1) are potential biomarkers for distinguishing HIV/TM-coinfected patients.

**Conclusions:**

This is the first report to provide a global proteomic profile of serum samples from HIV/TM-coinfected patients. Our data provide insights into the proteins that are involved as host response factors during infection. These data shed new light on the molecular mechanisms that are dysregulated and contribute to the pathogenesis of HIV/TM coinfection. IL1RL1 and THBS1 are promising diagnostic markers for HIV/TM-coinfected patients although further large-scale studies are needed. Thus, quantitative proteomic analysis revealed molecular differences between the HIV/TM-coinfected and HIV-mono-infected individuals, and might provide fundamental information for further detailed investigations.

**Electronic supplementary material:**

The online version of this article (10.1186/s12014-018-9219-8) contains supplementary material, which is available to authorized users.

## Background

Human immunodeficiency virus infection and acquired immune deficiency syndrome (HIV/AIDS) is a major public health problem leading to significant death rates worldwide, especially in developing countries including China. Since the beginning of the epidemic, more than 70 million people have been infected with HIV worldwide, and approximately half of these people are estimated to have died of AIDS-related causes [1, 2]. HIV infection leads to low levels of CD^4+^ T cells through various mechanisms, including pyroptosis of abortively infected T cells, apoptosis of uninfected bystander cells, direct viral killing of infected cells, and killing of infected CD^4+^ T cells by CD^8+^ cytotoxic lymphocytes that recognize infected cells [3–6]. When CD^4+^ T cell numbers decline below a critical level, cell-mediated immunity is lost, and the body becomes progressively more susceptible to a wide range of opportunistic infection, which may be caused by fungi, bacteria, viruses, and parasites that are normally controlled by the immune system [7–10]. Among these infections, fungal infections are a major cause of HIV-related mortality globally [11, 12].

Fungi contribute substantially to opportunistic infections in patients with late-stage HIV infection. *Talaromyces marneffei* (TM), formerly known as *Penicillium marneffei* (PM), is an emerging pathogenic fungus that can cause severe opportunistic infections, including life-threatening systemic infections in HIV-infected patients, in endemic regions of Southeast Asia [13, 14]. In some regions such as Hong Kong and southern China, TM infection has long been considered as one of the top three AIDS-defining opportunistic infections, alongside tuberculosis and cryptococcosis [15]. Similar to other opportunistic pathogens, infection with TM exacerbates deterioration of the immune response and accelerates AIDS progression, as indicated by high fever, weight loss, skin lesions, hepatosplenomegaly, lymphadenopathy, and respiratory and gastrointestinal abnormalities, however, the mechanism remains elusive [16, 17]. Recent data exploring the effects of TM infection on mortality in HIV/AIDS patients in China revealed that the mortality of TM-infected patients (25.0 per 100 person-months) was the highest among all HIVAIDS-associated complications and was significantly higher than that of TM-uninfected HIV/AIDS patients (13.8 per 100 person-months) [18]. Some data suggest that TM infection is common in hospitalized HIV/AIDS patients in Southeast Asia and associated with a higher mortality rate than most HIV-associated complications, but the mechanism remains elusive [13, 14, 18]. At present, early diagnosis of HIV/TM is considered to be key for effective treatment and prognosis. Overall, the diagnosis of HIV/TM still remains a major challenge due to the inadequacy of current diagnostic methods and the poor sensitivity and/or specificity of existing markers [6, 12, 16].

Through host-fungal pathogen interactions, fungi are known to regulate host macromolecular synthesis by modifying the host transcription and translation machineries and forcing the hosts to provide the requirements of the fungus during infection [6, 8, 10, 11, 19]. Some of these fungal requirements may lead to epigenetic modifications that are associated with a variety of biological processes, including cell differentiation, proliferation, and immunity. Therefore, host proteins that are involved in interactions with TM play essential roles in Talaromycosis (formerly Penicilliosis) progression across a spectrum of clinical syndromes that vary from cutaneous lesions to hepatosplenomegaly, fever, lymphadenopathy and ultimately mortality. Accumulated data showed that the initial interactions of the TM conidia with host phagocytic cells and the degree of activation of the host’s innate immune responses in response to the fungus are critical parameters determining the host’s ability to control the disease [20–22]. Using proteomic profiling, whole cell proteins from the early stages of mold to yeast development in TM were resolved by two-dimensional gel electrophoresis. Some differentially expressed proteins have been identified and categorized into the following groups: heat-shock responses, the catalase family, cell wall biosynthesis, amino acid metabolism, and energy metabolism [8, 23–25]. These results suggest that remarkably different proteins are involved in the molecular pathogenesis of HIV-related diseases between TM-uninfected and TM-infected patients.

Comparative proteomic approaches involving tandem mass tag (TMT) are widely used to analyze host responses in animals, humans, and plants during fungal infections [26–30]. In addition, the use of TMT quantitative proteomics to screen for diagnostic and prognostic protein biomarkers has also been reported [31, 32]. Therefore, clinical proteomic strategies provide an overall understanding of the host factors involved in fungal infection and provide insights into the alterations in signaling pathways, allowing us to further understand fungal pathogenesis. Serum proteomic analysis is a valuable approach for the discovery of protein biomarkers for the early recognition, diagnosis, monitoring and treatment of a disease, including fungal infections [22, 33–36]. Here, we examined the differential expression of proteins in pooled serum HIV/AIDS patients with TM infection using TMT-based quantitative proteomics. Our findings provide the first insights into the molecular differences between HIV/TM-coinfected and HIV-mono-infected patients, and might supply fundamental information for future research.

## Methods

### Patients and sample collection

The study population comprised inpatients who were identified as being serologically positive for HIV infection for the first time between January 2015 and December 2016 at the HIV inpatient unit of the Fuzhou Infectious Disease Hospital in Southeast China. All newly identified cases of HIV infection were confirmed by western blotting and reported to the Center for Disease Control (Fuzhou, China) [37]. TM was isolated and identified from skin lesions or tongues of HIV/AIDS patients and cultured on Sabouraud agar plates. Patients were excluded if they had previously received any antiretroviral therapy. The investigated population is depicted in Table 1.

**Table 1:** Baseline characteristics of the HIV/TM-coinfected and HIV-mono-infected individual groups in this study

Characters	Mono-infected group (n = 18)	Co-infected group (n = 18)	*p* value
Gender (M/F)	18/0	18/0	
Age (years)	40.08 ± 14.22	37.22 ± 9.18	0.216
Mean CD4 count (cells/μl)	97.05 ± 35.07	16.15 ± 12.58	0.029
Mean viral load (log_10_)	6.23 ± 1.64	6.72 ± 1.26	0.063

All serum samples were collected and processed according to standard operating procedures to minimize preanalytical variation [26, 27]. The serum samples from HIV/AIDS patients were divided into 2 groups: TM-negative (A) and TM-positive (B). For each group, every 6 individual samples with equal volumes of serum were mixed, and the pooled serum samples were then subjected to depletion of high-abundance proteins according to the manufacturer’s instructions (Agilent Technologies, Santa Clara, CA, USA). Six repeated protein extracts for each group were used to minimize the individual differences among patients. The use of human biopsy samples in this project was approved by the Institutional Review Board of Fuzhou Infectious Diseases Hospital of Fujian Medical University. Written consent was received from all participants in this study.

### Protein preparation and TMT labeling

The ProteoMiner™ Protein Enrichment Kit (Bio-Rad, Hercules, CA, USA) was utilized to optimize the decrease in abundant proteins according to the manufacturer’s instructions. The proteins were precipitated with cold 15% trichloroacetic acid (TCA) for 2 h at − 20 °C. After centrifugation at 4 °C for 10 min, the supernatant was discarded. The remaining precipitate was washed with cold acetone for three times. The proteins were redissolved in buffer (8 M urea, 100 mM TEAB, pH 8.0) and the protein concentration was determined with a 2-D Quant kit (GE Healthcare, Little Chalfont, UK) according to the manufacturer’s instructions.

For digestion, the protein solution was incubated with 10 mM dithiothreitol (DTT) for 1 h at 37 °C and then alkylated with 20 mM iodoacetamide (IAA) for 45 min at room temperature in the dark. For trypsin digestion, the protein sample was diluted by adding 100 mM triethylamonium bicarbonate (TEAB) to a urea concentration of less than 2 M. Finally, trypsin was added at a 1:50 trypsin-to-protein mass ratio for the first digestion overnight and a 1:100 trypsin-to-protein mass ratio for a second 4 h-digestion. Approximately 100 μg of protein for each sample was digested with trypsin for the following experiments.

After trypsin digestion, the peptide was desalted by a Strata X C18 SPE column (Phenomenex, CA, USA) and vacuum-dried. The peptide was reconstituted in 0.5 M TEAB and labeled with the TMT 6-plex™ Label Reagent set according to the manufacturer’s protocol for the 6-plex TMT kit (Thermo Fisher Scientific, Waltham, MA, USA). TMT 126, 127, 128, 129, 130 and 131 were used to label TM-negative samples (A) and TM-positive samples (B) (Additional file 1: Table S3). The reporter ion isotopic distributions (− 2, − 1, + 1, + 2) are primarily for carbon isotopes with reporter ion interference for each mass tag shown. Reporter ion isotopic distributions can be used as isotope correction factors in the TMT 6-plex method template in Proteome Discoverer software version 1.4 and above. Briefly, one unit of TMT reagent (defined as the amount of reagent required to label 100 μg of protein) was thawed and reconstituted in 24 μl of acetonitrile (ACN). The peptide mixtures were incubated with TMT reagent for 2 h at room temperature and then pooled, desalted and dried by vacuum centrifugation.

### Quantitative proteomic analysis by LC–MS/MS

The sample was then fractionated by high-pH reverse-phase high-performance liquid chromatography (HPLC) using an Agilent 300 Extend C18 column (5 μm particles, 4.6 mm ID, 250 mm length). Briefly, peptides were first separated into 80 fractions with a gradient of 2% to 60% acetonitrile in 10 mM ammonium bicarbonate, pH 10, over 80 min. Then, the peptides were combined into 18 fractions and dried by vacuum centrifugation.

The peptides were dissolved in 0.1% formic acid (FA) and directly loaded onto a reversed-phase precolumn (Acclaim PepMap 100, Thermo Scientific, USA). Peptide separation was performed using a reversed-phase analytical column (Acclaim PepMap RSLC, Thermo Scientific, USA). The gradient was comprised of increasing solvent B (0.1% FA in 98% ACN) from 8% to 26% in 22 min, 26% to 40% in 12 min and 40% to 80% in 3 min and then holding at 80% for the last 3 min, all at a constant flow rate of 400 μl/min on an EASY-nLC 1000 UPLC system. The resulting peptides were analyzed by a Thermo Scientific™ Q Exactive™ plus hybrid quadrupole-Orbitrap mass spectrometer (Thermo Fisher Scientific, Waltham, MA, USA).

The peptides were subjected to NSI source followed by tandem mass spectrometry (MS/MS) in Q Exactive™ plus (Thermo Fisher Scientific, Waltham, MA, USA) coupled with the UPLC. Intact peptides were detected in the Orbitrap at a resolution of 70,000. Peptides were selected for MS/MS using the NCE setting as 30; ion fragments were detected in the Orbitrap at a resolution of 17,500. A data-dependent procedure that alternated between one MS scan followed by 20 MS/MS scans was applied for the top 20 precursor ions above a threshold ion count of 1E4 in the MS survey scan with a 30.0 s dynamic exclusion. The electrospray voltage applied was 2.0 kV. Automatic gain control (AGC) was used to prevent overfilling of the Orbitrap; 5E4 ions were accumulated for the generation of MS/MS spectra. For MS scans, the m/z scan range was 350–1800. The fixed first mass was set as 100 m/z.

### Data analysis

The resulting MS/MS data were processed using MaxQuant with an integrated Andromeda search engine (v.1.5.2.8). Tandem mass spectra were searched against the SwissProt human database (20150806 Q: 20203). Trypsin/P was specified as a cleavage enzyme allowing up to 2 missing cleavages, 5 modifications per peptide and 5 charges. The mass error was set to 10 ppm for precursor ions and 0.02 Da for fragment ions. Carbamidomethylation on Cys was specified as a fixed modification, and oxidation on Met was specified as a variable modification. False discovery rate (FDR) thresholds for proteins, peptides and modification sites were specified at 1%. The minimum peptide length was set at 7. The minimum reporter PIF in the MaxQuant software was 0.75. For the quantification method, TMT 6-plex was selected. All the other parameters in MaxQuant were set to the default values. Identified protein domains were annotated by InterProScan (a sequence analysis application) based on the protein sequence alignment method using the InterPro (http://www.ebi.ac.uk/interpro/) domain database.

To identify the differentially expressed proteins, the relative protein expression values were compared between the HIV/TM-coinfected-(B) and HIV-monoinfected (A) patient groups. The proteins were considered to be differentially expressed if the TMT ratios were > 1.5 or < 0.67 in the serum samples of HIV/AIDS patients infected with TM compared to those from the TM-uninfected group, with a *p* value < 0.05, which was statistically analyzed by a paired *t* test. We used a volcano plot (shown in Fig. 2a) to display the differentially expressed proteins, where the x-axis represents the log_2_-based fold change, and the y-axis represents the negative log_10_ of the *p* value calculated from the two-tailed *t* test. The red points shown in the top left panel represent significantly upregulated proteins, while the green points represent significantly downregulated proteins.

### Functional analysis of the differentially expressed proteins

The Gene Ontology (GO) annotation proteome was derived from the UniProt-GOA database (http://www.ebi.ac.uk/GOA/). GO annotation contains three categories: biological process, cellular compartment, and molecular function. For each category, a two-tailed Fisher’s exact test was employed to test the enrichment of the differentially expressed protein against all identified proteins. Correction for multiple hypothesis testing was carried out using standard FDR control methods. GO terms with a corrected *p* value < 0.05 were considered significant.

The Kyoto Encyclopedia of Genes and Genomes (KEGG) database was used to annotate the protein pathways. First, we used the KEGG online service tool KAAS to annotate the protein’s KEGG database description. Then, we mapped the annotation result on the KEGG pathway database using the KEGG online service tool KEGG mapper.

### Immunoblotting analysis

The expression of the selected proteins in serum samples was verified by western blotting (WB), as previously reported [11, 19, 27]. Briefly, 30 µg of protein from each sample were separated by SDS-PAGE and transferred onto NC membranes (Millipore, Bradford, MA, USA). Then, the membranes were blocked for 2 h in PBST buffer containing 5% bovine serum albumin (BSA) and probed with IL1RL1 and THBS1 primary antibodies (1:1000 dilution, Santa Cruz Biotechnology, USA) and a β-actin antibody (1:5000 dilution, TransGen Biotech, China) at 4 °C overnight. After washing 3 times with PBST buffer for 10 min, the membranes were incubated with appropriate HRP-conjugated secondary antibodies (1:5000 dilution, TransGen Biotech, China) for 1 h at room temperature. Following another wash in TBST buffer, the protein expression levels were detected by enhanced chemiluminescence and visualized by autoradiography. Quantification of the western blot band intensity was performed using ImageJ 1.45 software (NIH) according to the manufacturer’s instructions.

### Statistical analysis

The data were expressed as the mean ± SD, and differences between two groups were analyzed with Student’s *t* test. *p* < 0.05 was considered statistically significant. Statistical analysis was performed using the Statistical Program for Social Sciences (SPSS) software 17.0 (SPSS Inc., Chicago, IL, USA).

## Results

### Clinical characteristics of the study population

A total of 36 serum samples from TM-positive and TM-negative (control) HIV/AIDS patients were used in this study (Table 1). All selected patients had similar ages and the same sex. However, compared to the patients in the TM-negative control group, the HIV/AIDS patients with TM infection had a significantly higher mean CD^4+^ T cell count (97.05 ± 35.07 vs. 16.15 ± 12.58, *p* = 0.029) and a higher HIV viral load (6.73 ± 1.64 vs. 6.22 ± 1.26, *p* = 0.063).

### Relative quantification of the serum proteome between the HIV/TM co-and HIV mono-infected individuals

**Fig. 1:**
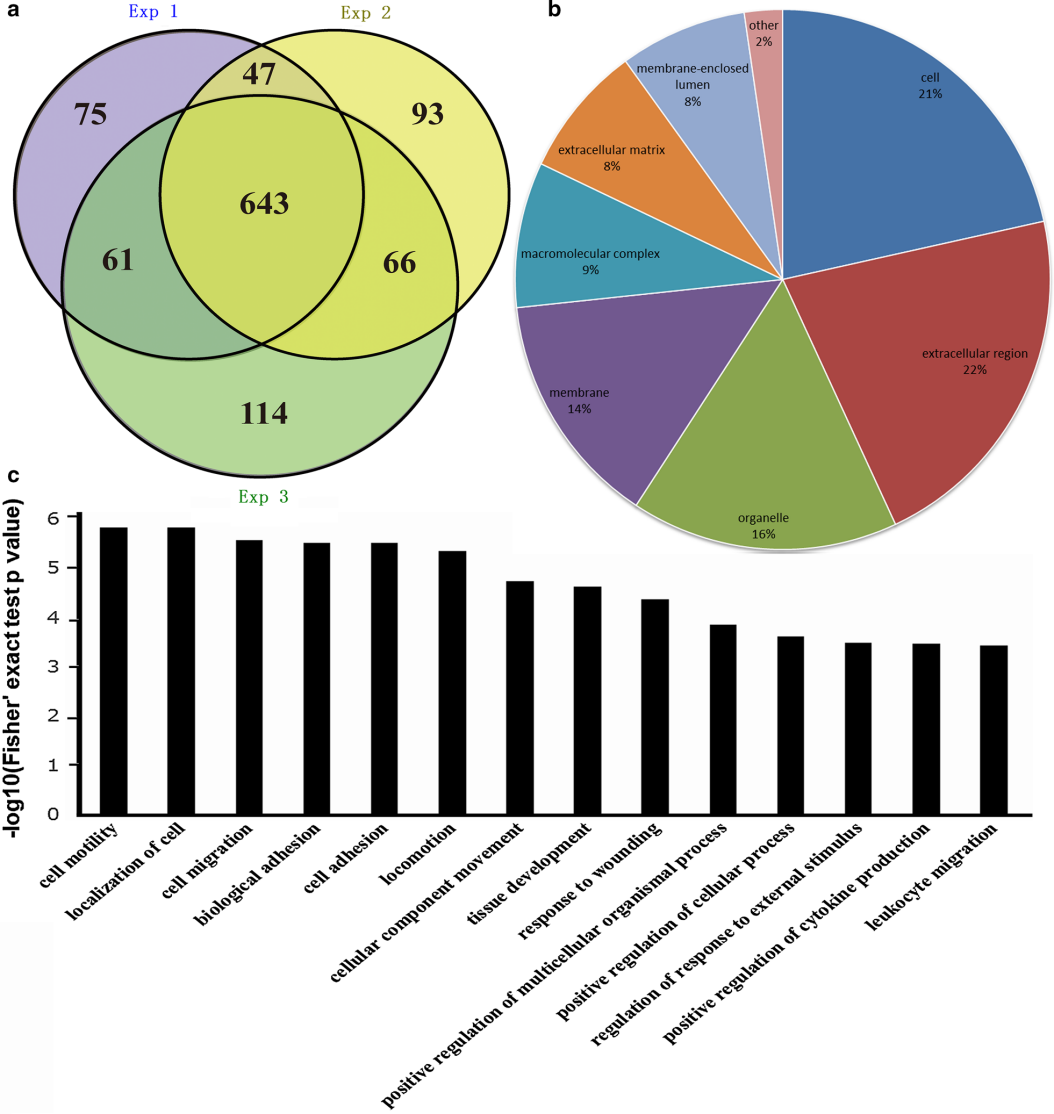
Features of the proteome data set of Venn diagrams and TMT-labelling shotgun analysis between the HIV/TM-coinfected-and HIV-mono-infected patient serum samples. **a** Venn diagrams shows the numbers of identified proteins and the overlaps of protein identification in 3 repeated experiments. **b** Subcellular localizations of the identified proteins. **c** GO analysis of the involved biological processes. The analysis was performed using DAVID and gene ontology annotations

Using the results from MaxQuant with an integrated Andromeda search engine (v.1.5.2.8), we quantified 826, 849, and 884 proteins in TMT 6-plex labeling replicates. Among the three replicates, 643 proteins overlapped, accounting for 58.51% of the total quantified proteins (Fig. 1a, Additional file 2). The overlapping proteins identified by MS were subsequently classified by bioinformatics analysis. GO annotation was applied to classify the identified proteins in terms of their subcellular localizations, and each protein was assigned at least one term. More than 20% of the proteins were annotated as belonging to extracellular and cell-associated proteins, and the other two main categories of these proteins were the organelle (16%) and membrane (14%) compartments (Fig. 1b). Although the extracellular, cell, organelle and membrane proteins were the most highly represented categories in the extracts, macromolecular complex and membrane-enclosed lumen proteins were also readily identified, which indicated that the protein extraction procedure was not strongly biased to a few cell compartments. As summarized in Fig. 1c, the top three molecular functions were associated with cell motility, cell localization, and cell migration.

### Differentially expressed proteins associated with TM infection

**Fig. 2:**
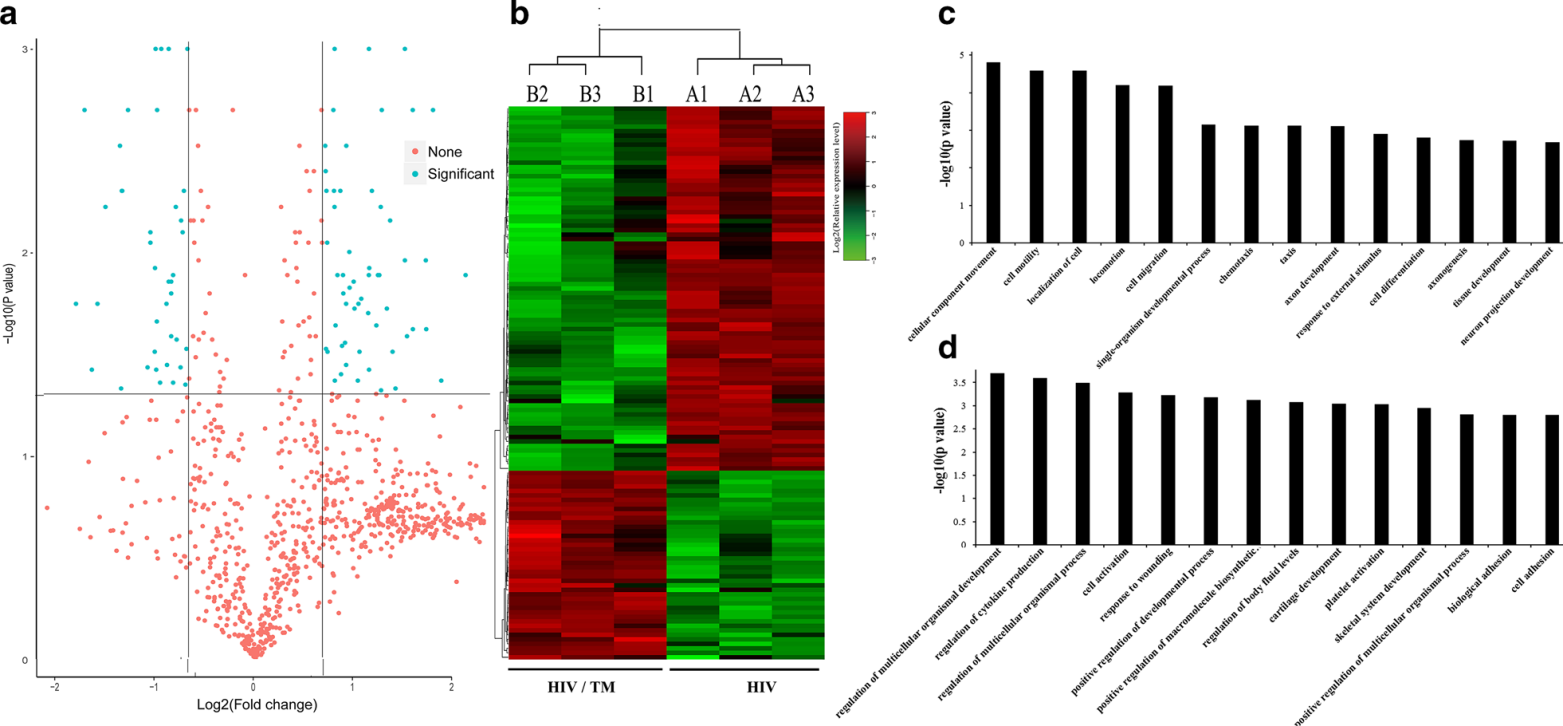
The hierarchical clustering and involved biological processes analysis of differentially expressed proteins between the HIV/TM-coinfected-and HIV-mono-infected patient serum samples. **a** Volcano plot represented the protein abundance changes (**b** vs. **a**). A total of 123 dysregulated proteins with fold change ≥ ± 1.5 and *p* values < 0.05 were identified. **b** Hierarchical clustering of the 123 dysregulated proteins (B vs. A). **c**, **d** GO analysis of the upregulated proteins (**c**) and downregulated proteins (**d**) involved biological processes in the HIV/TM co-and HIV mono-infected patient serum samples (**b** vs. **a**). Only terms with *p* values less than 0.001 are shown

To identify the differentially expressed proteins, a comparative analysis of relative protein expression values was conducted between the HIV/TM-coinfected and HIV-monoinfected patient groups. In this study, 123 proteins with a mean expression fold change of ≥ ± 1.5 (log_2_ 0.58) were identified as differentially expressed proteins in the serum of HIV/AIDS patients with TM infection compared to the TM-uninfected group (Fig. 2a, Additional file 2). When the ratio of these 123 proteins was plotted on a heatmap, 81 proteins were upregulated, while 42 proteins were downregulated in the serum samples between the HIV/TM-coinfected and HIV-monoinfected patient groups, and these two types of proteins formed distinct clusters (Fig. 2b). We further analyzed the involvement of these proteins in biological processes by GO analysis; the results showed that the 123 up-and downregulated proteins formed distinct clusters, and most of these dysregulated proteins were strongly associated with cell motility, activation and regulation (Fig. 2c, d). These results clearly proved that there might be different molecular backgrounds and molecular mechanisms between the TM-infected and TM-uninfected control groups.

### KEGG pathway analysis of the differentially expressed proteins

**Fig. 3:**
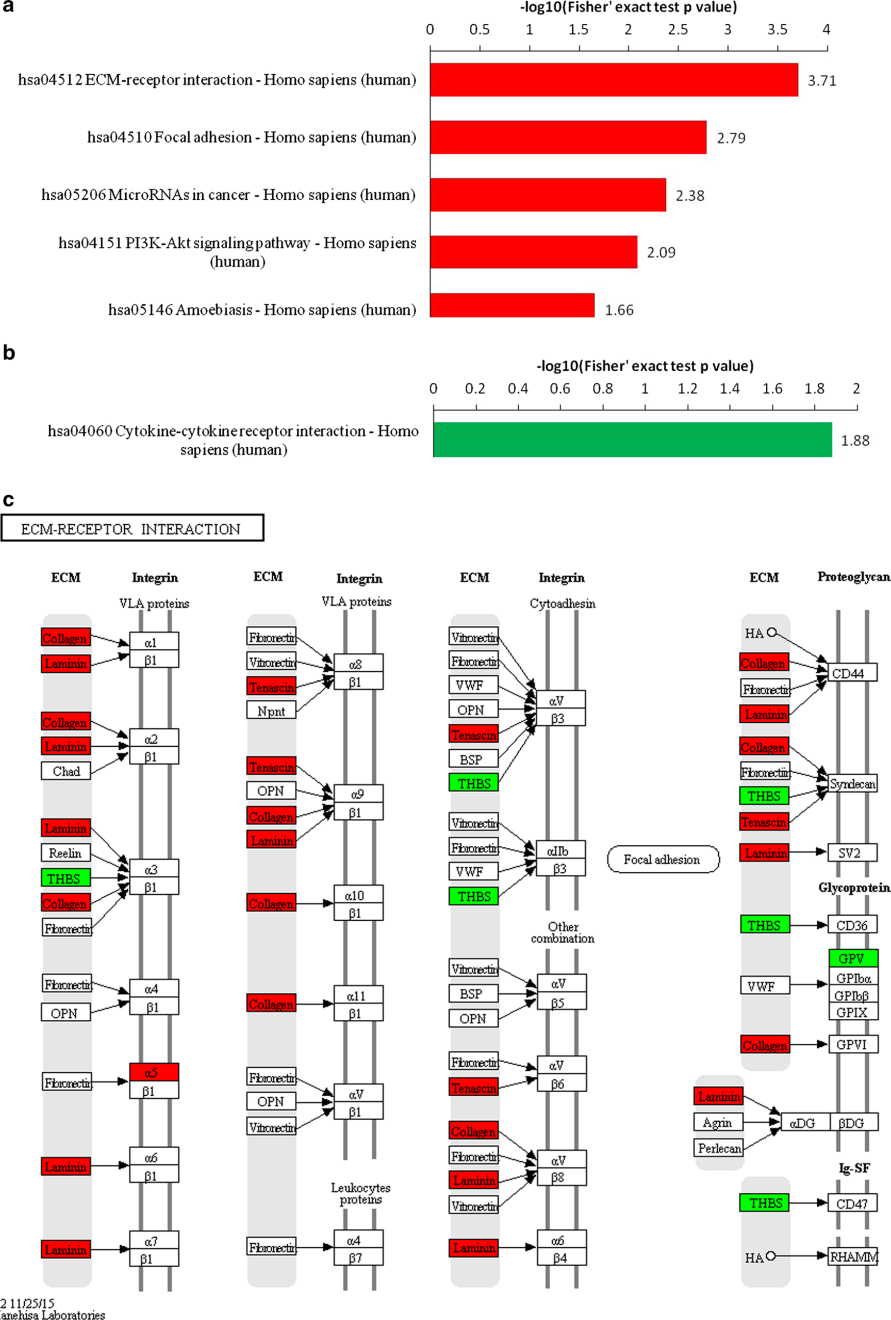
The key signaling pathways involved in the HIV/TM-coinfected-and HIV-mono-infected patient serum samples. KEGG pathway-based enrichment analysis of up-regulated (**a**) and down-regulated (**b**) proteins (**b** vs. **a**). **c** Pathway obtained from KEGG pathway analysis. The red labeling indicates the up-regulated proteins and green labeling indicates the down-regulated proteins

To further analyze the roles of the protein expression alterations between the HIV/TM-coinfected and HIV-monoinfected patients, interactive network analysis of the differentially expressed proteins was performed using the KEGG online service tool. In this work, KEGG generated several networks that illustrated strong hubs of dysregulation in TM-infected patients. As shown in Fig. 3b, the dysregulated proteins mainly participated in the regulation of the extracellular matrix (ECM)-receptor interaction, focal adhesion, microRNAs in cancer, the PI3K-AKT signaling pathway, amoebiasis and cytokine–cytokine receptor interactions. Of these categories, the ECM-receptor interaction pathway was the highest scoring KEGG network and had a significance score of 3.71 (Fig. 3a). Based on this network, 34 proteins were found to be differentially expressed in TM-positive HIV/AIDS patients compared with TM-negative controls. Among these proteins, 27 proteins displayed increased expression, and 7 proteins exhibited decreased expression, as shown in Fig. 3a. Therefore, TM infection is expected have its own specific molecular characteristics and molecular mechanisms.

### Verification of the differential expression of IL1RL1 and THBS1

According to hierarchal clustering analysis (Fig. 2) and KEGG network analysis (Fig. 3), the proteins Interleukin-1 receptor-like 1 (IL1RL1, 10.62-fold) and thrombospondin 1 (THBS1, 1.82-fold) were differentially expressed in HIV/AIDS patients between the TM-positive group and the TM-negative control group. IL1RL1, which is a member of the Toll-like receptor superfamily based on the structure of its intracellular Toll/interleukin-1 receptor (TIR) domain, has considerable prognostic value and is used to aid in risk stratification to identify patients who are at high risk of mortality and rehospitalization among patients diagnosed with heart failure [38–40]. THBS1, a subunit of a disulfide-linked homotrimeric protein, plays a functional role in inhibiting tumor growth, cell migration, and neovascularization and acts as an endogenous tumor suppressor by interacting with its receptors, CD36 and CD47, or activating transforming growth factor-beta signaling. Therefore, these factors are potentially interesting biomarkers to distinguish between HIV/TM-coinfected and HIV-monoinfected patients [41, 42]. Hence, the expression changes in IL1RL1 and THBS1 were further investigated by WB analyses using independent sets of 14 serum samples (7A and 7B).

**Fig. 4:**
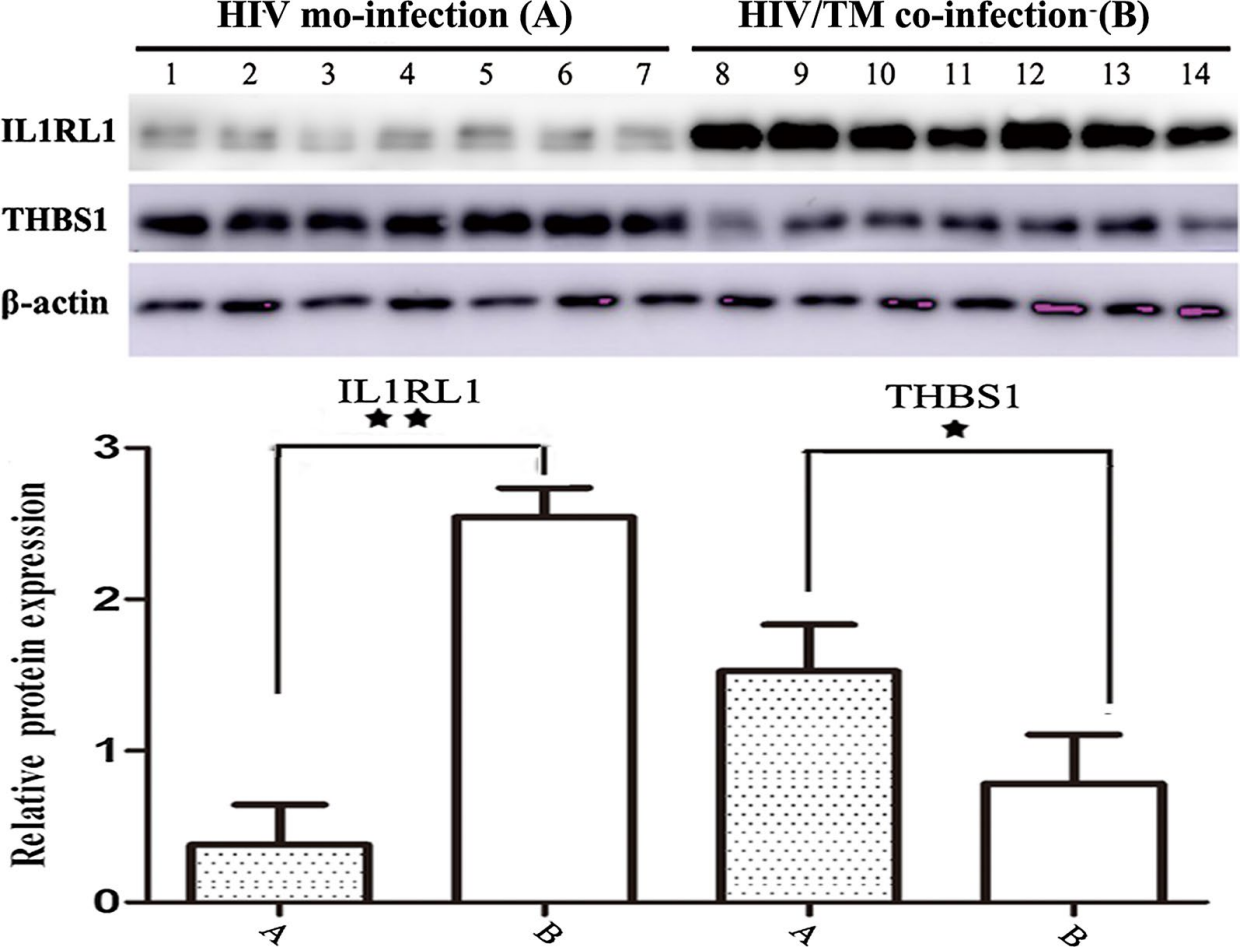
Validation of the differentially expressed proteins of IL1RL1 and THBS1 between the HIV/TM-coinfected-and HIV-mono-infected patient serum samples

Figure 4 shows that the protein expression level of IL1RL1 was significantly upregulated, while that of THBS1 was downregulated in HIV/AIDS patient serum samples with TM infection compared to the TM-negative group. The protein expression level of IL1RL1 was significantly upregulated by 9.6-fold (n = 14 patients, *p* < 0.01) in HIV/TM-coinfected patient serum samples compared to the HIV-monoinfected group. In contrast, the protein expression level of THBS1 was downregulated 1.8-fold (n = 14 patients, *p* < 0.05) in HIV/AIDS patient serum samples with TM infection compared to the TM-negative group.

Overall, we have successfully identified the TM infection-associated proteins IL1RL1 and THBS1 in HIV/AIDS patients, which is consistent with the LC–MS/MS results obtained in Figs. 1 and 2. Therefore, these two proteins might be potential biomarkers for distinguishing TM infection in HIV/AIDS patients, but the underlying molecular mechanisms need to be further dissected.

## Discussions

HIV/AIDS disease is currently and will continue to be a major worldwide health problem [6]. With HIV/AIDS patients coinfected with TM being responsible for a significant proportion of fatal systemic mycosis cases, the results of previous studies confirm that compared to HIV-monoinfected individuals, HIV/TM-coinfected patients are associated with an increased risk of developing opportunistic infections and cancers [2, 6, 13]. While much of the existing literature has focused on noting the presence of disparities between HIV/TM-coinfected and HIV-monoinfected patients, little is known about the potential mechanisms and the differences in specific biological pathways within the context of TM infection. The proteomic data presented in this study are the first to report differences between HIV/TM-coinfected and HIV-monoinfected patients at the proteome level. These data not only confirm earlier studies that demonstrated that HIV/TM-coinfected patients might have a poorer prognosis than patients without TM infection but also provide new information regarding HIV/TM-coinfected patients, which will facilitate further detailed investigations.

In this study, we carried out a quantitative proteomic analysis of serum samples from HIV/AIDS patients coinfected with TM and uninfected controls using TMT labeling and LC–MS/MS to quantify the dynamic changes in the whole serum proteome of HIV/AIDS patients. Here, 949 proteins were quantified, of which 123 proteins were differentially expressed by > 1.5- or < 0.67-fold. Bioinformatics analysis showed that most of the differentially expressed proteins were involved in the single-organism process, response to stimulus, cellular process, biological regulation, and metabolic process, which mainly reflected inflammatory disorders and abnormal functions of innate immunity. For example, compared to patients without TM infection, HIV/TM-coinfected patients have a higher risk of inflammatory disorders and poorer clinical outcomes; therefore, HIV-infected patients with TM have a higher incidence of developing acute or persistent pulmonary disease, disseminated infection, and reactivation disease [14, 16]. Furthermore, this phenomenon was further supported by the obviously altered ECM-receptor interaction pathway in HIV/AIDS patients in the TM-positive and TM-negative groups, which influences tissue/organ morphogenesis and the maintenance of cell/tissue structure and function. A significant discovery from this work was that many genes related to the ECM-receptor interaction pathway may be used as novel biological markers of HIV/TM-coinfected patients [43]. The ECM contains many large molecules, such as collagens, elastin, microfibrillar proteins, and proteoglycans. In addition to being necessary structural components, ECM molecules also play critical roles in the control of key cellular events, such as adhesion, migration, differentiation, proliferation, and apoptosis [43].

Our analysis also revealed that the differentially expressed protein IL1RL1, which has been proven to be a valuable biomarker of cardiac stress and a strong predictor of cardiovascular death and the risk of developing new heart failure in syncytiotrophoblast elevation myocardial infarction (STEMI) and non-ST elevation acute coronary syndrome (NSTE-ACS) patients [38, 39], was the most upregulated protein (fold change = 10.62) in serum samples from HIV/TM-coinfected patients compared to HIV mono-infected patients [38, 39]. Different studies have indicated that IL1RL1 is increased in severe asthma and is associated with multiple indicators of TH2-like inflammation, including blood eosinophils, exhaled nitric oxide, epithelial CLCA1 and eotaxin-3 [40]. In this study, upregulation of IL1RL1 expression may be dissected into independent signals with distinct functional consequences for the pathway that is central to disease pathogenesis in HIV/TM-coinfected patients. The specific mechanism needs to be clarified in future studies.

In addition, the expression of THBS1 was significantly downregulated in the serum of HIV/TM-coinfected patients compared to that in the serum of HIV-monoinfected patients. THBS1 was initially described as a potent angiogenic inhibitor that acts mainly through regulating the secretion of VEGF to limit the angiogenesis process, but it also plays several other roles in the response to inflammation, promoting the resolution of the inflammatory process and facilitating the phagocytosis of damaged cells [41]. In patients with cancer, there is dysregulation of angiogenesis control, with upregulation of VEGF and downregulation of TSP1, favoring tumor angiogenesis and inflammation [41, 42]. Consistent with the results of KEGG network analysis suggesting that the ECM-receptor interaction pathway plays an important role in tissue and organ morphogenesis and in the maintenance of cell and tissue structure and function, many proteins associated with those functions, including THBS1, were dysregulated in our data. The ECM-receptor interaction pathway may contribute to the involvement of TM infection in various cellular activities through regulating the expression of many proteins, such as THBS1. Consistent with this idea, our observations indicated that THBS1 downregulation may decrease the activation of the ECM-receptor interaction pathway in serum samples from HIV/TM-coinfected patients compared to that in serum samples from HIV-monoinfected patients. Therefore, the expression level of THBS1 might be a potential biomarker for distinguishing HIV/TM-coinfected and HIV-monoinfected individuals. These serum biomarkers may aid in the diagnosis of HIV/TM coinfection, which can play an important role in disease control and clearance. However, the underlying mechanism by which THBS1 inhibits ECM-receptor interactions remains unclear.

Overall, we profiled serum proteins from HIV/TM-coinfected and HIV-monoinfected patients and identified two potential biomarkers and possible therapeutic targets for HIV/TM-coinfected and HIV-monoinfected individuals.

## Conclusions

Here, we applied the TMT method followed by mass spectrometric analysis to study the serum proteomic profile alterations between HIV/TM-coinfected and HIV-monoinfected patients. As expected, our results clearly proved that different protein profiles and signaling pathways were involved in HIV/TM-coinfected and HIV-monoinfected patients. To verify these results, the different expression levels of IL1RL1 and THBS1 in HIV/TM-coinfected and HIV-monoinfected patient samples were investigated by WB. In addition, the results of the present study suggested the potential application of the IL1RL1 and THBS1 proteins as biomarkers for distinguishing HIV/TM-coinfected and HIV-monoinfected individuals.

## Additional files

**Additional file 1: Figure S1.** Overall technical route of this project. Figure S2. Reproducibility analysis of this project. Figure S3. QC validation of MS data. (A) Mass error distribution of all identified peptides, (B) Peptide length distribution. Figure S4. Distribution of quantification results (at least 1.5-fold up-or downregulated). Figure S5. Protein domain enrichment analysis of upregulated (A) and downregulated (B) proteins (B vs A). Table S3. TMT-Labeling information. Table S4. Baseline Characteristics of the HIV/TM-coinfected-and HIV-mono-infected individual groups in verification samples.

**Additional file 2.** Protein list detected by LC–MS/MS. (Table S1, sheet 1) at least 1.5-fold up-or downregulated and (Table S2, sheet 2) total.

## Abbreviations

HIV/AIDS: human immunodeficiency virus infection and acquired immune deficiency syndrome
TM: *Talaromyces marneffei*
PM: *Penicillium marneffei*
TMT: tandem mass tag
TCA: trichloroacetic acid
DTT: dithiothreitol
IAA: iodoacetamide
TEAB: triethylamonium bicarbonate
ACN: dcetonitrile
HPLC: high performance liquid chromatography
LC–MS/MS: liquid chromatography mass separation-mass spectra characterization
FA: formic acid
AGC: automatic gain control
FDR: false discovery rate
GO: gene ontology
KEGG: kyoto encyclopedia of genes and genomes
ECM: extracellular matrix
PI3K-AKT: phosphatidylinositol 3′-kinase Protein kinase B
IL1RL1: interleukin-1 receptor-like 1
THBS1: thrombospondin 1
TIR: toll/interleukin-1 receptor
STEMI: syncytiotrophophoblast elevation myocardial infarction
NSTE-ACS: non-ST elevation acute coronary syndrome

## References

[1] Auld AF, Shiraishi RW, Oboho I, Ross C, Bateganya M, Pelletier V (2017). Trends in prevalence of advanced HIV disease at antiretroviral therapy enrollment-10 countries, 2004–2015. MMWR Morb Mortal Wkly Rep.

[2] Lucas S, Nelson AM (2015). HIV and the spectrum of human disease. J Pathol.

[3] Doitsh G, Galloway NL, Geng X, Yang Z, Monroe KM, Zepeda O (2014). Cell death by pyroptosis drives CD4 T-celldepletion in HIV-1 infection. Nature.

[4] Garg H, Mohl J, Joshi A (2012). HIV-1 induced bystander apoptosis. Viruses.

[5] Gizzi AS, Grove TL, Arnold JJ, Jose J, Jangra RK, Garforth SJ (2018). A naturally occurring antiviral ribonucleotide encoded by the human genome. Nature.

[6] Limper AH, Adenis A, Le T, Limper AH, Adenis A, Le T (2017). Fungal infections in HIV/AIDS. Lancet Infect Dis.

[7] Chu C, Pollock LC, Selwyn PA (2017). HIV-associated complications: a systems-based approach. Am Fam Physician.

[8] Lau SKP, Tsang CC, Woo PCY (2017). *Talaromyces marneffei* genomic, transcriptomic, proteomic and metabolomic studies reveal mechanisms for environmental adaptations and virulence. Toxins (Basel).

[9] Samaranayake LP, Fidel PL, Naglik JR, Sweet SP, Teanpaisan R, Coogan MM (2002). Fungal infections associated with HIV infection. Oral Dis.

[10] Armstrong-James D, Meintjes G, Brown GD (2014). A neglected epidemic: fungal infections in HIV/AIDS. Trends Microbiol.

[11] Wei D, Li NL, Zeng Y, Liu B, Kumthip K, Wang TT (2016). The molecular chaperone GRP78 contributes to toll-like receptor 3-mediated innate immune response to hepatitis C virus in hepatocytes. J Biol Chem.

[12] Chang CC, Megan C, Zhou JL, Michael M, Post JJ, Cameron BA (2013). HIV and co-infections. Immunol Rev.

[13] Stathakis A, Lim KP, Boan P, Lavender M, Wrobel J, Musk M (2015). *Penicillium marneffei* infection in a lung transplant recipient. Transpl Infect Dis.

[14] Vanittanakom N, Cooper CR, Fisher MC, Sirisanthana T (2006). *Penicillium marneffei* infection and recent advances in the epidemiology and molecular biology aspects. Clin Microbiol Rev.

[15] Hu Y, Zhang J, Li X, Yang Y, Zhang Y, Ma J (2013). *Penicillium marneffei* infection: an emerging disease in mainland China. Mycopathologia.

[16] Yan Q, Li Y, Wan L, Tian R, Guo Q, Li S (2011). *Penicillium marneffei*-stimulated dendritic cells enhance HIV-1 trans-infection and promote viral infection by activating primary CD^4+^ T cells. PLoS ONE.

[17] Chen R, Ji G, Ma T, Huang X, Ren H, Xi L (2015). Role of intracellular free calcium in killing *Penicillium marneffei* within human macrophages. Microb Pathog.

[18] Jiang J, Meng S, Huang S, Ruan Y, Lu X, Li JZ (2018). Effects of *Talaromyces marneffei* infection on mortality of HIV/AIDS patients in southern China: a retrospective cohort study. Clin Microbiol Infect.

[19] Wei D, Zhang X (2010). Proteomic analysis of interactions between a deep-sea thermophilic bacteriophage and its host at high temperature. J Virol.

[20] Li GC, Zhang L, Yu M, Jia H, Tian T, Wang J (2017). Identification of novel biomarker and therapeutic target candidates for acute intracerebral hemorrhage by quantitative plasma proteomics. Clin Proteomics.

[21] Sapmak A, Kaewmalakul J, Nosanchuk JD, Vanittanakom N, Andrianopoulos A, Pruksaphon K (2016). *Talaromyces marneffei* laccase modifies THP-1 macrophage responses. Virulence.

[22] Kumar GS, Venugopal AK, Mahadevan A, Renuse S, Harsha HC, Sahasrabuddhe NA (2012). Quantitative proteomics for identifying biomarkers for tuberculous meningitis. Clin Proteomics.

[23] Xi L, Xu X, Liu W, Li X, Liu Y, Li M (2007). Differentially expressed proteins of pathogenic *Penicillium marneffei* in yeast and mycelial phases. J Med Microbiol.

[24] Chandler JM, Treece ER, Trenary HR, Brenneman JL, Flickner TJ, Frommelt JL (2008). Protein profiling of the dimorphic, pathogenic fungus, *Penicillium marneffei*. Proteome Sci.

[25] Lau SK, Tse H, Chan JS, Zhou AC, Curreem SO, Lau CC (2013). Proteome profiling of the dimorphic fungus *Penicillium marneffei* extracellular proteins and identification of glyceraldehyde-3-phosphate dehydrogenase as an important adhesion factor for conidial attachment. FEBS J.

[26] Sinclair J, Timms JF (2011). Quantitative profiling of serum samples using TMT protein labelling, fractionation and LC-MS/MS. Methods.

[27] Song SH, Han M, Choi YS, Dan KS, Yang MG, Song J (2014). Proteomic profiling of serum from patients with tuberculosis. Ann Lab Med.

[28] Park EC, Lee SY, Yun SH, Choi CW, Lee H, Song HS (2018). Clinical proteomic analysis of scrub typhus infection. Clin Proteomics.

[29] Gerold G, Meissner F, Bruening J, Welsch K, Perin PM, Baumert TF (2015). Quantitative proteomics identifies serum response factor binding protein 1 as a host factor for hepatitis C virus entry. Cell Rep.

[30] Wei D, Zeng Y, Xing X, Liu H, Lin M, Han X (2016). Proteome differences between hepatitis B virus genotype-B-and genotype-C-induced hepatocellular carcinoma revealed by iTRAQ-based quantitative proteomics. J Proteome Res.

[31] Zhao C, Wang F, Wang P, Ding H, Huang X, Shi Z (2015). Early second-trimester plasma protein profiling using multiplexed isobaric tandem mass tag (TMT) labeling predicts gestational diabetes mellitus. Acta Diabetol.

[32] Gao W, Xu J, Wang F, Zhang L, Peng R, Shu Y (2015). Plasma membrane proteomic analysis of human gastric cancer tissues: revealing flotillin 1 as a marker for gastric cancer. BMC Cancer.

[33] Yang Y, Huang J, Rabii B, Rabii R, Hu S (2014). Quantitative proteomic analysis of serum proteins from oral cancer patients: comparison of two analytical methods. Int J Mol Sci.

[34] Xu DD, Deng DF, Li X, Wei LL, Li YY, Yang XY (2014). Discovery and identification of serum potential biomarkers for pulmonary tuberculosis using iTRAQ-coupled two-dimensional LC–MS/MS. Proteomics.

[35] Wei L, Wang Y, Lin L, Zhang L, Shi Y, Xiang P (2018). Identification of potential serum biomarkers of acute paraquat poisoning in humans using an iTRAQ quantitative proteomic. RSC Adv.

[36] Aruni AW, Zhang K, Dou Y, Fletcher H (2014). Proteome analysis of coinfection of epithelial cells with Filifactor alocis and *Porphyromonas gingivalis* shows modulation of pathogen and host regulatory pathways. Infect Immun.

[37] Chen Y, Wang Z, Huang A, Yuan J, Wei D, Ye H (2016). A trend towards increasing viral load in newly diagnosed HIV-infected inpatients in southeast China. Epidemiol Infect.

[38] Gordon ED, Palandra J, Wesolowska-Andersen A, Ringel L, Rios CL, Lachowicz-Scroggins ME (2016). IL1RL1 asthma risk variants regulate airway type 2 inflammation. JCI Insight.

[39] Vocca L, Di Sano C, Uasuf CG, Sala A, Riccobono L, Gangemi S (2015). IL-33/ST2 axis controls Th2/IL-31 and Th17 immune response in allergic airway diseases. Immunobiology.

[40] Grupper A, AbouEzzeddine OF, Maleszewski JJ, Grupper A, Geske JR, Kremers WK (2018). Elevated ST2 levels are associated with antibody mediated rejection in heart transplant recipients. Clin Transpl.

[41] Zhao Y, Xiong Z, Lechner EJ, Klenotic PA, Hamburg BJ, Hulver M (2014). Thrombospondin-1 triggers macrophage IL-10 production and promotes resolution of experimental lung injury. Mucosal Immunol.

[42] Matsuo Y, Tanaka M, Yamakage H, Sasaki Y, Muranaka K, Hata H (2015). Thrombospondin 1 as a novel biological marker of obesity and metabolic syndrome. Metabolism.

[43] Baeten KM, Akassoglou K (2011). Extracellular matrix and matrix receptors in blood–brain barrier formation and stroke. Dev Neurobiol.

