# Supplementary material for: Proteomic analysis of serum proteins from HIV/AIDS patients with Talaromyces marneffei infection by TMT labeling-based quantitative proteomics
Source: Clin Proteomics. 2018 Dec 21;15:40. doi: 10.1186/s12014-018-9219-8 (PMC6302400; doi:10.1186/s12014-018-9219-8)
Supplement: Supplementary file 1 — Additional file 1: Figure S1. Overall technical route of this project. Figure S2. Reproducibility analysis of this project. Figure S3. QC validation of MS data. (A) Mass error distribution of all identified peptides, (B) Peptide length distribution. Figure S4. Distribution of quantification results (at least 1.5-fold up-or downregulated). Figure S5. Protein domain enrichment analysis of upregulated (A) and downregulated (B) proteins (B vs A). Table S3. TMT-Labeling information. Table S4. Baseline Characteristics of the HIV/TM-coinfected-and HIV-mono-infected individual groups in verification samples. [file 12014_2018_9219_MOESM1_ESM.docx]

**Additional file 2**

Proteomic analysis of serum proteins from HIV/AIDS patients with *Talaromyces marneffei* infection by TMT labeling-based quantitative proteomics

Yahong Chen^12^, Aiqiong Huang^12^, Wen Ao^12^, Zhengwu Wang^12^, Jinjin Yuan^12^, Qing Song^5^, Dahai Wei^1234^ * and Hanhui Ye ^12^*

^1^Mengchao Hepatobiliary Hospital of Fujian Medical University, Fuzhou 350025, P. R. China

^2^Fuzhou Infectious Disease Hospital of Fujian Medical University, Fuzhou 350025, P. R. China

^3^The First Affiliated Hospital of Jiaxing University, Jiaxing 314001, P. R. China

^4^Department of Immunology and Microbiology, Shanghai Jiao Tong University School of Medicine, Shanghai 200025, P. R. China

^5^Shanxi Institute of Flexible Electronics, Northwestern Polytechnical University, Xi'an 710072, P. R. China

* Corresponding Authors (correspondence should be addressed to DH. Wei and HH. Ye), Postal Address: Xihong Road 312, Fuzhou 350025, Fujian Province, P.R. China,

Tel.: 86-591-83705927

Fax: 86-591-83705927

E-mail addresses: weidahai3166@hotmail.com, yehanhui@163.com

**Running title:** Proteomic analysis of serum proteins with *Talaromyces marneffe*

**Supplementary Figures:**

**
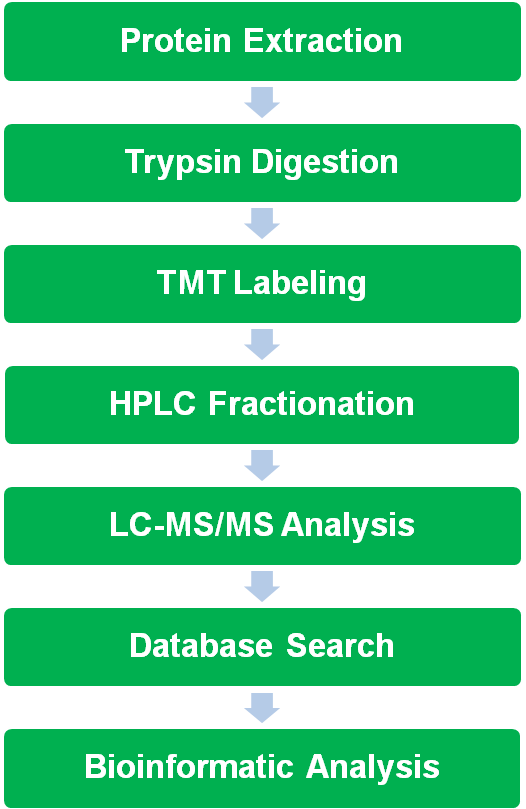
**

Figure S1. Overall technical route of this project.

**
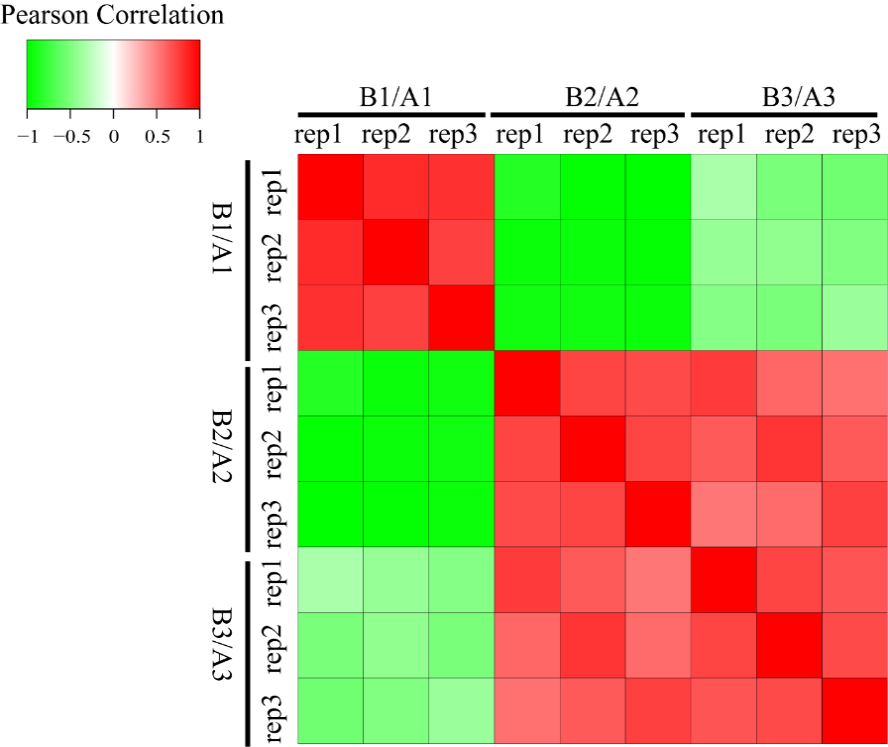
**

Figure S2. Reproducibility analysis of this project.


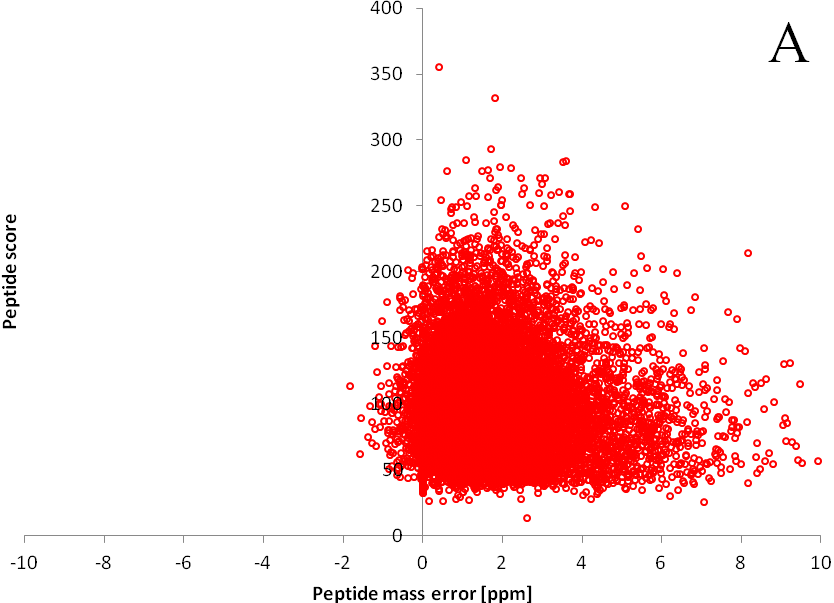


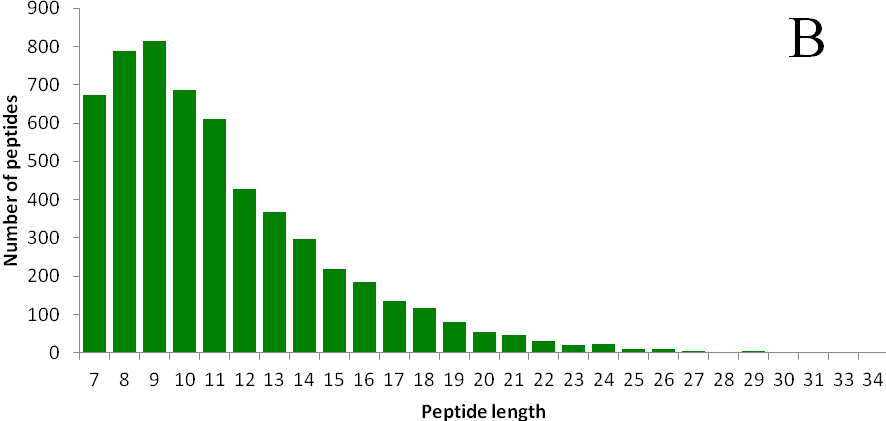


Figure S3. QC validation of MS data. (A) Mass error distribution of all identified peptides, (B) Peptide length distribution.


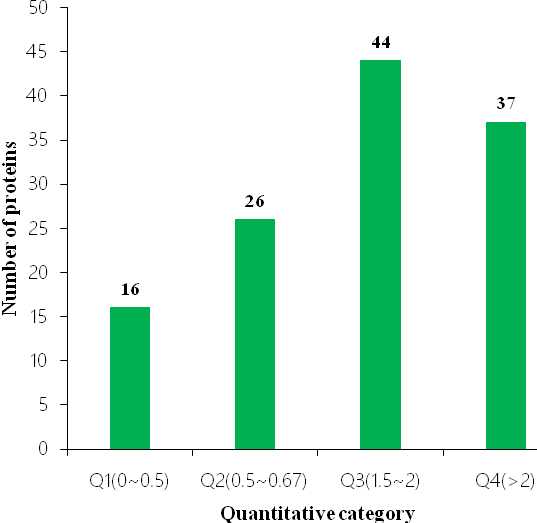


Figure S4. Distribution of quantification results (at least 1.5-fold up- or down-regulated).


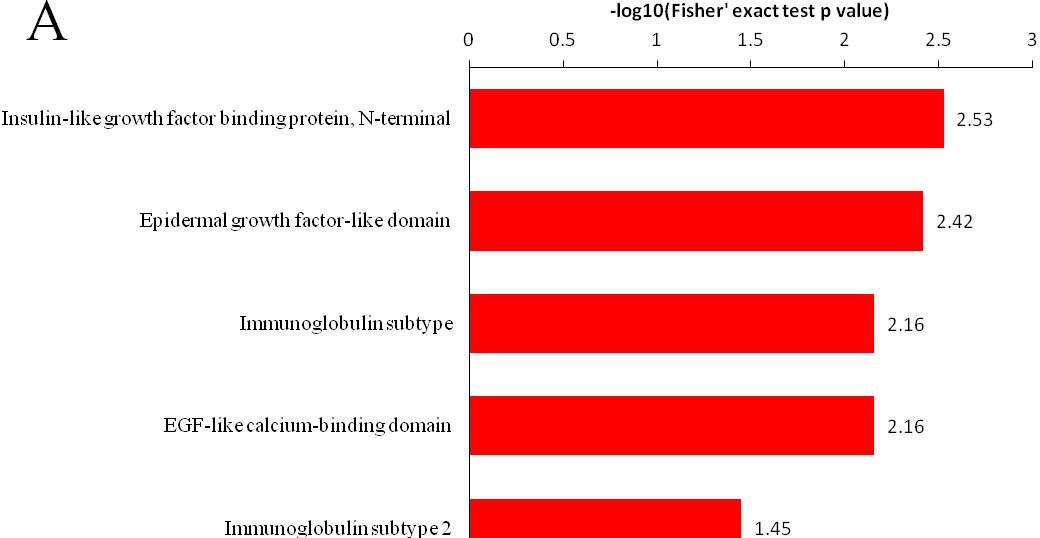


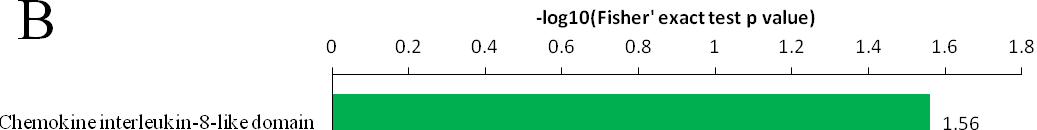
 Figure S5. Protein domain enrichment analysis of up-regulated (A) and down-regulated (B) proteins (B vs A).

Table S3. TMT Labeling information (Reporter Ion Isotopic Distributions)

| **Mass Tag** | **Mass Reporter** | **Reporter Ion** | **-2** | **-1** | **Monoisotopic** | **1** | **2** | **Sample Groups** |
| --- | --- | --- | --- | --- | --- | --- | --- | --- |
| TMT^6^-126 | 126 | 126.127726 | 0.0% | 0.0% | 100% | 6.7% | 0.2% | A1 |
| TMT^6^-127 | 127N | 127.124761 | 0.0% | 0.4% | 100% | 5.3% | 0.2% | A2 |
| TMT^6^-128 | 128C | 128.134436 | 0.0% | 1.6% | 100% | 4.9% | 0.0% | A3 |
| TMT^6^-129 | 129N | 129.131471 | 0.0% | 1.6% | 100% | 3.9% | 0.0% | B1 |
| TMT^6^-130 | 130C | 130.141145 | 0.1% | 2.9% | 100% | 2.5% | 0.0% | B2 |
| TMT^6^-131 | 131 | 131.138180 | 0.2% | 3.3% | 100% | 2.3% | 0.0% | B3 |

Table S4. Baseline Characteristics of the HIV/TM-coinfected- and HIV-mono-infected individual groups in verification samples

| **Characters** | **Mono-infected group (n=18)** | **Co-infected group (n=18)** | ***P* value** |
| --- | --- | --- | --- |
| Gender (M/F) | 7/0 | 7/0 |  |
| Age (years) | 40.16±13.01 | 37.91±8.82 | 0.291 |
| Mean CD4 count (cells/μl) | 92.33±30.12 | 22.24±11.92 | 0.032 |
| Mean viral load (log_10_) | 6.37±1.55 | 6.81±1.31 | 0.102 |
|  |  |  |  |
